# In vitro properties of concentrated canine platelets stored in two additive solutions: a comparative study

**DOI:** 10.1186/s12917-017-1236-8

**Published:** 2017-11-15

**Authors:** N. Hlavac, C. S. Lasta, M. L. Dalmolin, L. A. Lacerda, D. de Korte, N. A. Marcondes, S. R. Terra, F. B. Fernandes, F. H. D. González

**Affiliations:** 1 Clinical Pathology Laboratory, Veterinary Medicine Faculty, Universidade do Sul de Santa Catarina, Tubarão, Brazil; 2 Veterinary Medicine Faculty, Centro Universitário Ritter dos Reis – Laureatte International Universities, Porto Alegre, Brazil; 3 Blut’s Diagnosis Center and Veterinary Services, Porto Alegre, Brazil; 4 Sanquin Blood Bank and Sanquin Research, Sanquin, Amsterdam, the Netherlands; 5 Post-Graduation Program in Medicine: Medical Sciences, Universidade Federal do Rio Grande do Sul, Porto Alegre, Brazil; 6 Zanol Laboratory, Porto Alegre, Brazil; 7 Department of Veterinary Clinical Pathology, Universidade Federal do Rio Grande do Sul, Porto Alegre, Brazil

**Keywords:** Composol, Platelet storage lesion, SSP +, Storage solutions, Veterinary transfusion medicine

## Abstract

**Background:**

Platelet transfusion therapy poses many challenges in veterinary clinical practice. Lack of readily available blood donors, short shelf-life, and inability to administer a sufficient number of platelets to meet a dog’s transfusion need are the major difficulties encountered. Platelet additive solutions are already in use at American and European human blood banks, showing to be a realistic alternative. This study compares the in vitro platelet function in plasma, Composol, or SSP+ during storage for 13 days. Platelet rich plasma-platelet concentrate with 35% plasma and 65% platelet additive solutions (Composol or SSP+) and a control group (100% plasma) were prepared. Swirling, platelet count, blood gases, metabolic variables, platelet activation markers, and apoptosis markers were analyzed on days 1, 5, 9 and 13.

**Results:**

Swirling was well preserved and pH was acceptable (> 6.2) during storage for all platelet additive solutions units until day 9. SSP + units showed more stable pH and metabolic variables until day 13. Platelets in plasma showed higher glucose consumption than in Composol or in SSP+. The platelet additive solutions units showed better platelet metabolism maintenance, reduced glucose consumption and lactate production. The apoptotic markers were still low for 9 days in platelet concentrates with platelet additive solutions, suggesting the possibility to extend the shelf life with the use of SSP+ or Composol.

**Conclusions:**

Our findings suggest that the uses of Composol and SSP+ in canine platelet concentrates are potential alternatives in veterinary blood banks.

**Electronic supplementary material:**

The online version of this article (10.1186/s12917-017-1236-8) contains supplementary material, which is available to authorized users.

## Background

One of the goals of transfusion medicine is to assure the production of quality blood components. The platelet concentrate (PC) availability is limited due to its short-term storage. During storage, platelets suffer structural and biochemical changes that are collectively called platelet storage lesions (PSL) [1]. Extending the shelf-life has effects on the quality of platelets which have been studied in PC stored for more than 5 days. For several reasons, storing platelets in platelet additive solutions (PAS) is turning into a routine practice, and it is already standard in human blood banks in Europe and The United States [2].

PAS are isotonic crystalloid media containing citrate as anticoagulant and acetate as fuel for aerobic metabolism. Initially, the main motivation to use PAS was to increase the availability of plasma for the fractioning and production of derived components. However, other advantages were observed, such as being a sterile environment free of pathogens and the standardized composition in comparison to plasma from donors [3–5].

Several PAS formulations are being tested since 1980. In vivo studies with the most recently developed solutions have shown good results of platelet increment and recovery as well as the reduced occurrence of transfusion reactions in human patients [6–8].

Studies in human blood banks have already tested the platelet metabolism in vitro using PAS for periods exceeding 12 days [9, 10]. In some countries in Europe, the storage timing of PC was extended to 7 days with the use of PAS [2]. There are already four PAS generations, Composol (PAS-D) and SSP+ (PAS-E), tested in this study, are from the third generation and are used to preserve PC in European blood banks [3, 6, 11].

The search for new additive solutions for PC preservation has always been a concern in human medicine, and nowadays it is also of interest for the veterinary medicine. The biochemical evaluation during canine PC storage with different PAS represents some practical information for veterinary medicine. Canine platelet metabolism has similar characteristics to the human, and the PC storage time is also a difficulty at the veterinary blood banks. The quality control requirements of canine PC are based on human models. Therefore, it is believed that the results concerning the viability and PSL should be similar to the already reported in vitro tests done with human PC.

This study aims to compare the parameters of in vitro platelet metabolism from concentrates of canine platelets stored in plasma and in two PAS.

## Methods

A volume of 450 mL whole blood was collected in blood bag with CPD^1^ from healthy canine donors, weighing over 28 kg. Physical examination, CBC, chemistry profile, and infectious disease screening were performed for each dog. The animals were privately owned; all owners provided their consent for their dogs to be used in this study in accordance with bioethics concepts applied to animal research. This study was approved by the Ethics Committee on Animal Use from the Federal University of Rio Grande do Sul (Approval protocol #20528).

The experiment was carried out in three groups (Treatment 1: 100% plasma (control) *n* = 13; Treatment 2: SSP+^2^ *n* = 13; Treatment 3: Composol^3^ *n* = 14), in a total of 40 blood bags.

### PC preparation

After collection, the bags were left undisturbed at room temperature for one hour and then were processed to obtain the PC through the platelet rich plasma method (PRP). The first centrifugation^4^ was light, 1600 *g* for 6 min at 22 °C.

The PRP was transferred to an empty TOTM-PVC^1^ bag through manual plasma extractor.^5^ The PRP bag was subjected to a second spin^4^ (3300 *g* for 8 min at 22 °C), to remove the excess of plasma. Manual extraction^5^ was used and additive solutions^2,3^ were added in the test groups in the proportion of 65% of PAS to 35% of residual plasma as recommended by the manufacturer's (32–47%) [12], with a final volume of about 65 mL (22 ± 2 mL of plasma and 42 ± 2 mL of PAS). The control group PCs (100% plasma) were prepared in the traditional way, by manual extraction^5^ targeting the volume of approximately 65 mL. The compositions of the chosen PAS are shown in Additional file 1.

A sampling site coupler^6^ was placed in all bags [13]. The calculated volume of PAS was added with the aid of sterile syringe and needle. The volume of the units was calculated in accordance with the specific gravity of resuspension solution (1.026 g/mL for plasma, 1.006 g/mL PAS) [3].

After this procedure, the bags were identified and kept at rest for an hour. Afterward, they were placed in a linear platelet shaker^7^ located in a preservation chamber^8^ in regulated temperature (22–24 °C) for 13 days.

### Sampling

The PCs stored in each treatment were subjected to evaluation at established time points. The sampling was done by sampling site coupler^6^ using syringe and sterile needles [14].

Analyzes were carried out on days 1, 5, 9 and 13. Platelet counts, MPV, PDW, swirling, pH, glucose, lactate, LDH, pO_2_ and pCO_2_, ATP, and flow cytometry to evaluate CD61, CD62P, Annexin V and JC-1 were assessed. Residual leukocyte count was performed on day 1 (24 h after collection). PCs samples were sent for microbiological culture on days 5 and 13.

### Qualitative and morphological variables

Before the bag sampling, an evaluation of platelet swirling was performed. Swirling is a non-invasive method for assessing platelet viability; it is caused by light diffraction due to the alignment of normal discoid shaped platelets. In this evaluation zero indicates no swirling and 3 indicates great swirling [15]. Residual leukocyte count was performed using the Nageotte chamber.^9^ The MPV, PDW and platelet counts were performed on an automated hematology counter.^10^

### Surface markers and platelet function

Identification of platelet surface markers was performed through flow cytometry with monoclonal antibodies (MoAbs) CD61 FITC,^11^ used for identification and quantification of platelet population; and p-selectin^12^ (CD62P), for evaluation of platelet activation.

### Gas analysis and metabolic variables

To assess platelet metabolism, the values of bicarbonate, glucose, pO_2_ and pCO_2_ were determined through a portable gas analyzer.^13^ Also, in order to determine the ATP, samples were prepared in trichloroacetic acid solution, frozen at −80 °C [12], and measured by bioluminescence assay^14^ aided by multi-mode microplate reader,^15^ according to the manufacturer’s instructions. The determination of lactate was performed through dry chemistry.^16^ The pH was measured by pH meter^17^ according to the manufacturer’s instructions. All samples were tested in duplicate.

### Markers of apoptosis and mitochondrial potential

LDH was analyzed with dry chemistry^16^. The exposure of PS (phosphatidylserine) was assessed with annexin V marker^18^ and the percentage of cells positive for the marker was quantified. Alterations in mitochondrial membrane potential (ΔΨm) were determined with JC-1^19^evaluation. Relatives degrees of mitochondrial polarization were quantified by measuring the red-shifted (FL-2) JC-1 aggregates, which are favored under of high membrane potential.

All flow cytometric experiments were performed on a flow cytometer^20^ where 30,000 events were acquired for each analysis^20^. Data were analyzed using FCS Express 5 software.^21^

### Microbiological analysis

Inoculation was performed in BHI, with aerobic and anaerobic culture at 37 °C in samples from days 5 and 13 of assessment.

### Statistical analysis

The quantitative data were expressed as the mean ± standard deviation. Two-way ANOVA was performed to analyse the effects of PAS and plasma. One-way ANOVA was performed to analyse the residual WBC count. When indicated, a post-hoc Tukey test or Duncan’s multiple range test were performed. The analyses were performed through Graph Pad Prism 6.0.^22^ The results with *p* < 0.05 were considered significant. Pearson correlation between variables was performed, considering significant values of *p* < 0.05.

## Results

### Composition of platelet concentrates

In vitro quality parameters were evaluated on day 1 (Table 1). All PCs had leukoreduction, average volume, pH, swirling and platelet concentration according to the quality control recommendations of regulatory authorities [16], and there was no significant difference between treatments. Three bags were excluded due to a contamination by *Staphylococcus spp*. negative coagulase on days 5 (one from each PAS) and 13 (Composol unit).

**Table 1:** Residual leukocyte, platelet counts, swirling and pH on the experimental day 1

	Day 1		
	Plasma	SSP+	Composol
Volume (ml)	65.92 ± 1.977	64.05 ± 2.66	63.48 ± 3.11
Residual leukocytes (×10^6^/unit)	0.0037 ± 0.0021	0.010 ± 0.009	0.005 ± 0.002
Platelet count (×10^10^/unit)	7.197 ± 2.86	7.88 ± 2.13	7.64 ± 2.44
Swirling	3 ± 0	3 ± 0	2.86 ± 0.53
pH (22 °C)	7.162 ± 0.24 a	6.72 ± 0.20 b	6.44 ± 0.45 b

Different lowercase letters represent significantly different values (*p* < 0.05) between treatments. Results reported as mean ± standard deviation

### In vitro evaluation during the stock

**Fig. 1:**
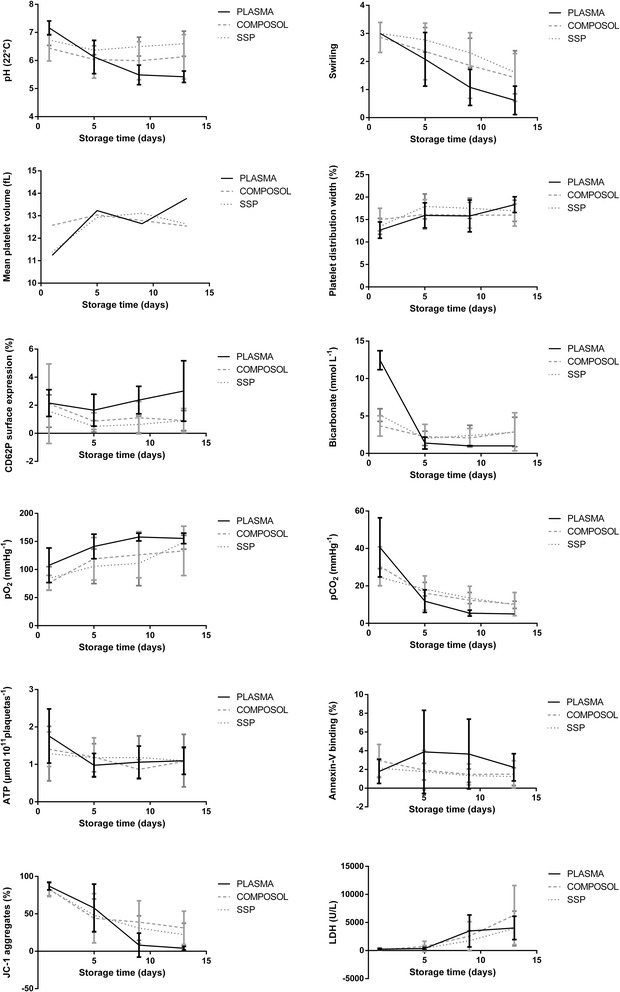
Linear regression graphs of the in vitro parameters of plasma platelet concentrates and additive solution, stored for 13 days

Platelet count remained stable over storage time and there was no difference between plasma and PAS groups (Additional file 2). Regarding the swirling*,* a significant reduction in this parameter was observed over time (*p* < 0.001). The swirling was kept 2+ (suitable for use) until day 9 in both PAS, and until day 13 in the PC stored with SSP+ (Fig. 1).

The PDW and MPV morpho-structural rates showed differences between the days of evaluation. Both parameters had a significant increase in days 5, 9 and 13 compared to day 1 (*p* < 0.001) (Fig. 1), and there was a significant PDW increase in the control group on day 13 (*p* < 0.001) compared to the PAS groups.There was no significant difference in platelet count and labeling with CD61 along time or between groups. The percentage of CD61 positive cells remained above 85% (Additional file 2). The platelet activation evidenced by CD62P was higher in the control group compared to PAS (*p* < 0.001), with no difference among the PAS groups. There was an increased CD62P on day1 (*p* = 0.014) in all treatments, and on day 13 in the control group (*p* < 0.001).

On day 1, the control group showed higher pH values in comparison to the PAS groups (Fig. 1), however, the control group showed significant decrease along the storage time. Meanwhile in Composol and SSP+ there was an increase of pH during long-term storage. In Composol solution, pH was below the acceptable (< 6.2) on days 5, 9 and 13. The units held in SSP+ showed a more stable pH during the 13 days of storage (Fig. 1, Additional file 2).

**Fig. 2:**
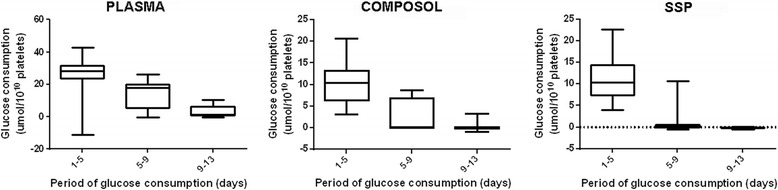
Glucose consumption rates of platelet concentrates stored in plasma and additive solution for 13 days

**Fig. 3:**
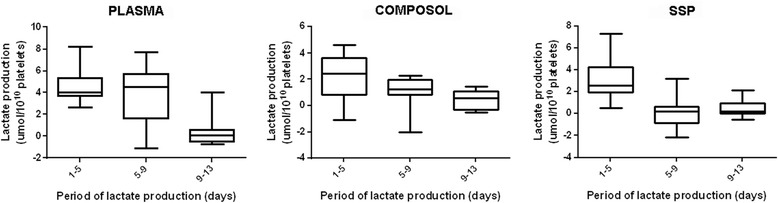
Lactate production rates of platelet concentrates stored in plasma and additive solution for 13 days

A decrease in ATP concentration was observed in all groups, there was no difference between the treatments (Fig. 1). A decline of glucose consumption and an increased lactate production along the time was observed (*p* < 0.001), and the average of consumption/production was significantly higher in the control group compared to the PAS groups (*p* < 0.001) (Figs. 2 and 3, Additional files 3 and 4).

There was a decrease in bicarbonate concentration during the storage time (*p* < 0.001). The initial bicarbonate concentration was higher in the control group (*p* < 0.001), the decrease in concentration was consequently higher in this group compared to PAS (Fig. 1). A reduction in pCO_2_ was observed during the storage time (*p* < 0.001) and the pO_2_ behaved inversely over the storage time in all groups (*p* = 0.001)(Fig. 1).

There were no alterations in PS throughout the storage time, and there was a higher expression in the control group compared to SSP+ (*p* = 0.007) and Composol (*p* = 0.050) (Fig. 1). The LDH activity showed an increase over time in all groups (Fig. 1), being significant from the 9th day of storage, without differences between the groups (*p* < 0.001) (Fig. 1).

Regarding the ΔΨm, there was a decrease in the percentage of JC-1 aggregates over the storage time (*p* < 0.001), leveling of between days 9 and 13. In the last 2 days of assessment, there was a significant lower percentage of JC-1 aggregates PC in the control group (*p* < 0.001) (Fig. 1).

### Correlation study

The results of the correlations (Table 2) emphasize the findings described before, and are closely related to aerobic and anaerobic metabolism performed by platelets in the stock period. A decrease in glucose, pCO_2_, HCO_3_, ATP, pH and ΔΨm was observed along time; and the data show a positive correlation between these parameters. Also, a positive correlation interposes between the lactate production, cytoplasmic LDH release, increase of pO_2_ and increased morpho-structural parameters - MPV and PDW.

**Table 2:** Pearson correlation between the metabolic characteristics of the stored plasma concentrates and the additive solution for 13 days

Correlations	Glucose	Lactate	pH	HCO_3_	ATP	pO_2_	pCO_2_	MPV	PDW	ΔΨm (JC-1)	LDH
Glucose	–	−.220*	.468**	.667**	.246*	.093	.338**	−.283*	−.321**	.284**	−.226*
Lactate	−.220*	–	−.663**	−.590**	−.285**	.596**	−.659**	.477**	.500**	−.800**	.463**
pH	.468**	−.663**	–	.706**	.253**	−.525**	.542**	−.549**	−.455**	.660**	−.227**
HCO_3_	.667**	−.590**	.706**	–	.397**	−.366**	.695**	−.507**	−.526**	.604**	−.073
ATP	.246*	−.285**	.253**	.397**	–	−.169*	.262**	−.160	−.128	.350**	−.099
pO_2_	.093	.596**	−.525**	−.366**	−.169*	–	−.736**	.256**	.257**	−.602**	.179*
pCO_2_	.338**	−.659**	.542**	.695**	.262**	−.736**	–	−.314**	−.389**	.708**	−.336**
MPV	−.283*	.447**	−.549**	−.507**	−.160	.256**	−.314**	–	.855**	−.440**	.017
PDW	−321**	.500**	−.455**	−.526**	−.128	.257**	−.389**	.855**	–	−.550**	.102
ΔΨm (JC-1)	.284**	−.800**	−.660**	.604**	.350**	−.602**	.708**	−.440**	−.550**	–	−.392**
LDH	−.226*	.463**	−.277**	−.073	−.099	.179*	−.336**	.017	.102	−.392**	–

*Correlation with significance level *p* < 0.05. **Correlation with significance level *p* < 0.01

## Discussion

This is the first study to assess the feasibility to store canine platelets with PAS obtained by PRP method. The comparative study of PAS revealed that both solutions maintained the in vitro quality of canine platelets for up to 9 days after collection. Parameters such as swirling, pH and platelet counts were maintained for up to 13 days with the use of SSP+.

The results of metabolic evaluation, cell death, mitochondrial membrane polarization and activation support the in vitro stability evidenced in this study. The control group also remained stable until the fifth day, as expected, except for the average pH value (6.1 ± 0.59). The values recommended by regulatory authorities and studies in humans and canines were taken as a comparative basis. When canine PC parameters in plasma and PAS – produced by PRP method – were compared, similar data to those produced by buffy-coat method (BC) or apheresis in humans were observed. However this is the first study that report PC viability in PAS produced by PRP method (Table 3) [3, 14, 16–22].

**Table 3:** Comparison of this experiment with other studies data reporting in vitro parameters of CP in plasma, SSP + and Composol - using 35% of residual plasma for the PAS - in different processing methods, plastic bags material and storage times

Assessed data	Plasma					SSP+		Composol		
% residual plasma	100%	100%	100%	100%	100%	35%	35%	35%	35%	35%
PC method	PRP	PRP	PRP	PRP	PRP	PRP	BC	BC	BC	PRP
N	64	8*	22*	15*	13*	13*	35	23	10	14*
PC storage conteiners	DEHP-PVC	DEHP-PVC	TOTM-PVC	TOTM-PVC	TOTM-PVC	TOTM-PVC	Varied	Varied	Polyolefin	TOTM-PVC
Leukoreduction	Yes	No	No	No	No	No	Yes	Yes	Yes	No
Storage time (days)	5	7	7	9	5	9	8	8	9	9
pH (day 5)	6.7 ± 0.26	7.30 ± 0.3	6.7 ± 0.2	> 7.0	6.1 ± 0.59	6.36 ± 0.15	> 6.6	> 6.6	6.93 ± 0.12	6.04 ± 0.67
Swirling (day 5)	> 2	Uninformed	> 2	Uninformed	2.07 ± 0.95	2.76 ± 0.43	Appropriate		2.0 ± 0.7	2.35 ± 1
Reference	Singh et al., 2009 [22]	Hoareau et al., 2014 [46]	Costa et al., 2011 [47]	Sink, 2002 [48]	This study		van der Meer et al., 2010 [49]		Van der meer, 2001 [50]	This study

*Data from studies with dogs

All PAS require residual plasma to metabolism maintenance. The experiences of American and European studies indicate that when using PAS with less than 20% residual plasma through the BC method, there will be difficulty in obtaining blood component with the desired quality. Therefore, researchers suggest a residual plasma volume of 32–47% [8, 11, 12]. Our study showed adequate platelet parameters using 35% of residual plasma with manual extraction technique, suggesting that the technique is feasible and easy to execute.

Adequate platelet count is one of the most important pieces of information for clinicians, so it is a crucial point when producing a PC [23]. Studies that evaluated canine PC in 100% plasma observed a significant reduction of platelet count, it was associated with platelet activation and formation of microaggregates, fragmentation or loss of platelet integrity, and these may be the reasons of reduction of platelet count during the storage period [20, 21]. Our results are in agreement with previous studies evaluating human PC in SSP+ and Composol, where no variation in platelet count was observed [3, 15, 19]. Van der Meer (2012) stated difference in this parameter, the authors suggest that the low viscosity of the PAS, makes it necessary to adapt and standardize the centrifugation protocols [24, 25].

Platelet swirling is used as a feasibility indicator to measure alterations in platelet morphology from discoid to spherical [12]. Corroborating the results of our experiment, other authors observed the maintenance of swirling with SSP+ and Composol for 7 days or longer (Table 3) [3, 11, 26].

In accordance with human PC data, alterations in MPV were not observed according to the used solution, this suggests that the change of format occurs regardless of the PAS used [7]. It is observed that the PDW in the control group has significant increase on the 13th day of storage which shows that the platelet fragmentation is more expressive in this group. These findings suggest that there is lysis and microparticle formation within the storage time [27, 28]. MPV showed a strong correlation with the pH decreasing over storage time, that indicate a loss of discoid to spherical shape, reflected in the MPV increase. Also we observed a strong correlation MPV and PDW with ΔΨm decreasing suggesting mitochondrial swelling and loose of potential (Fig. 1, Table 2) [29].

As a marker of platelet surface, the results of the use of CD61 are in agreement with other studies where the percentage of expression remained independent from the PAS used and the storage time [30, 31]. The CD62P is described with inconsistent results when correlating the increased expression with post-transfusion increment [29, 32]. In our experiment, the highest percentage of activation on day 1 was associated with the intense manipulation of the blood bag for its preparation, as described for human platelets. On day 13, CD62P expression was correlated with loss of platelet function [30].

When inferring the platelet metabolism, pH is one of the most used tools for quality control, turning into a mandatory assessment item at blood banks. When evaluating SSP+ and Composol with 35% of residual plasma in human PC, the pH maintenance was observed (>6.6) until the 8th day of storage (Table 3) [3, 30]. In agreement with previous studies, the pH decreased in all canine PC units during storage, but it remained within acceptable limits (> 6.2) until day 13 just in the samples stored in SSP+. In other studies, this solution obtained pH above 7.0 on the 7th day of storage, using similar amounts of residual plasma (30%) [11, 31].

The choice of TOTM-PVC material as plastic for the bags of this study was based on results of previous studies which showed better pH stability in canine PC when compared to the use of DEHP-PVC [20]. Studies using DEHP-PVC bags consequently showed a lower pH during storage [33, 34]. Both materials are recommended for human PC storage in Brazilian blood banks [35].

The reduction of ATP levels observed in our study was correlated to a decrease of glucose and ΔΨm which results in the reduction of energy metabolism. When metabolically active, the platelet mitochondria produce ATP through anaerobic glycolysis which occurs in the cytosol as well as through the aerobic oxidation that occurs by the tricarboxylicacid cycle (TCA) [8, 36]. In reference to metabolism, it is highlighted that the PAS have low amount of glucose in order to reduce the rate of anaerobic glycolysis, which can be clearly evidenced by glucose consumption and lactate production rates. In comparison to the control group, the variation in lactate production in PAS occurs because of their emphasizing the aerobic oxidation. In our study, the results of the lactate and glucose rates refer to the same behavior of human PCs evaluated in plasma or PAS [37, 38]. Glucose consumption and lactate production are also stimulated by the presence of phosphate in the storage media. Therefore, the 100% plasma group showed significantly higher conversion rate compared to Composol or SSP+. Composol, which contains no phosphate in its formulation, showed the lowest conversion rates. The phosphate is present in SSP+, but the presence of potassium and magnesium counteracts its effect, and this provides low conversion rates similar to those observed with Composol [39]. There are no studies evaluating production and consumption rates in canine PC, but the studies that assessed glucose and lactate concentration observed metabolic behavior and values similar to those reported in humans [20, 21].

The bicarbonate concentration is influenced by the composition of PAS [39]. It is also observed that the bicarbonate concentration was more stable in SSP+; this pattern reflects the most stable pH values in this PC group. The same behavior was observed in another study comparing 100% plasma PC with 70% SSP+ for 14 days [38]. The presence of bicarbonate in the plasma has a buffering effect and in order to compensate the absence of this component, acetate was added to PAS. In TCA, acetate is metabolized to acetyl-CoA, consuming H+, and oxidized to CO_2_ and H_2_O, acting in a buffering mode against pH reduction caused by glycolysis [6, 36, 40].

A reduction in pCO_2_ is observed along the storage time and pO_2_ behaves inversely. Studies in human PC report an increase of pO_2_ and a decrease in pCO_2_ associated with the decline of oxidative metabolism [34]. Similar results to our study were obtained when comparing plasma to PAS PC in humans, and correlated pCO_2_ values with the bicarbonate concentration of the medium [17, 30].

A previous study on PSL showed that the shelf-life and viability correlates with apoptosis mechanisms [41]. Therefore, the identification of intrinsic path markers (PS exposure, caspase marking) as well as extrinsic ones (quantification of cytochrome c, ΔΨm) have been used. The PS exposure is considered to be an activation marker with pro-coagulant activity, and also an apoptosis indicator [1, 32]. In our study, no alterations during storage time were observed, however there is a greater expression in the control group on all the days of evaluation if compared to SSP+. In most studies, the expression percentage increases along time in 100% plasma PC and with the use of PAS, with a more significant expression in plasma [3, 38]. One experiment evaluated in vitro canine platelets for 8 days and observed an increase of PS only after the addition of apoptosis inducing agent in the environment. Previously to the effect of the inducing agent, the expression percentage was similar to that observed in canine PC from our study [42]. Our experiment partially reproduces the behavior observed in human PC, although there is no increase over time, we observed a greater expression in the 100% plasma group. This scenario may reflect a limitation of the technique used in dogs; or the fact that the exposure of PS is not a good marker of recent apoptosis in canine platelets.

LDH is a marker of senescence. As the metabolic potential of platelets is exhausted, platelet lysis occurs as well as there lease of cytosolic components including LDH [10, 32]. Studies in canine and human plasma PC and the use of PAS in human PCs demonstrate results that corroborate with those observed in our experiment [19, 20, 30]. This mechanism also reflects the positive results of Pearson correlation with lactate and negative results with pH and ΔΨm (Fig. 1, Table 2).

The assessment of mitochondrial potential is most commonly used when the goal is to assess interference of mitochondria in storage time or situations when the PC is not kept under constant stirring. There is few data concerning mitochondrial function and platelet viability with the use of PAS [29, 43]. The results presented here are in accordance with the evaluation of this parameter using SSP+ in human PC [38] and a single study in canine plasma PC (Lasta C.: Metabolism of canine platelets stored as platelet concentrate for 5 days, unpublished). Another group assessed Composol in human PC, observing mitochondrial viability from 12 days of storage, the results were consistent with our study; where there is a drop of ΔΨm from the 9th day, which is more significant in the PC control group [10].

The bacterial contamination of 3 units was an obstacle. Coagulase negative *Staphylococcus* usually originates from the donor’s skin and is often isolated in PC; and when it is transfused, it can cause fatal reactions associated with endotoxemia. Because of the intense manipulation, the care must be redoubled when the additive solution is added in the processing [5]. Two of the excluded units were from donors in which the owner did not allow trichotomy. The authors suggest that in these cases, the bags do not be used for CP production. However, it was not possible to determine if the contamination occurred at the time of the blood collection or during the sampling procedure of the blood bags. The contaminated samples were excluded from the study.

### Correlations

The correlations enabled the identification of the main tendency among the variables used for platelet evaluation during storage. Platelet metabolism requires some direct correlations such as those between glucose and lactate, where the consumption of glucose directly results in lactate production, as well as between pH and pCO_2_, where the pH was negatively correlated with pCO_2_. Furthermore, the relationship between pO_2_ and pH suggests that oxygen availability is strongly associated with maintenance of pH in an osmotic balanced environment. Some correlations are indirect, but they are intuitive between glucose/lactate and morpho-structural rates (i.e. MPV, PDW), apoptotic markers (i.e. LDH) and ΔΨm. These parameters represent the measure of platelet viability; low glucose or no glucose is usually the result of prolonged storage, which indirectly results in loss of viability. The main cause for the consumption of glucose, besides the regular metabolism, is the constant energy requirement to maintain the osmotic balance and the integrity of the platelet membrane, lost over time. This metabolic shift to aerobic glycolysis is probably responsible for the observed correlations between glucose and lactate (Warburg effect). Immune cells, as platelets, switch their energy production from oxidative phosphorylation to glycolysis upon cell activation. This metabolic programming has been attributed to facilitate cytoskeletal changes, increased ion signaling, enhanced phospholipid turnover, and greater macromolecule synthesis in a very short time during platelet activation [44]. There is a strong correlation among glucose, lactate, MPV, LDH and ΔΨm which indicates that the metabolic rate is related to degranulation, osmotic destabilization and/or swelling.

## Conclusions

SSP+ and Composol seem to be an excellent alternative to replace plasma in the production of canine PC. Based on in vitro variables, canine PC storage in Composol is viable for 9 days, whereas the viability extends to 13 days with the use of SSP+. Prolonged storage, with in vitro quality maintenance, seems to be possible in veterinary medicine, with results similar to those seen in human platelets.

In vitro studies do not necessarily predict post-transfusion efficacy. Therefore, in vivo studies are needed to estimate the real contribution of PAS in preventing the PSL. This study provides a technical point of view needed for canine PC production with low plasma concentrations, and addition of PAS.

## Additional files

Additional file 1: Composition of the solutions used in the experiment. (DOCX 14 kb)

Additional file 2: Platelet count. pH and CD61 percentage of positive cells mean ± standard deviation. Different lowercase letters represent significantly different values (*p* < 0.05) between treatments. Different symbols represent significantly different values (*p* < 0.05) between assessment days. (DOCX 15 kb)

Additional file 3: Glucose consumption mean ± standard deviation of platelet concentrates stored in plasma and additive solution for 13 days. Different lowercase letters represent significantly different values (*p* < 0.05) between treatments. Different symbols represent significantly different values (*p* < 0.05) between assessment days. (DOCX 13 kb)

Additional file 4: Lactate production mean ± standard deviation of platelet concentrates stored in plasma and additive solution for 13 days. Different lowercase letters represent significantly different values (*p* < 0.05) between treatments. Different symbols represent significantly different values (*p* < 0.05) between assessment days. (DOCX 14 kb)

## Abbreviations

ATP: Adenosine-5′-triphosphate
BC: Buffy-coat method
BHI: Brain-heart infusion medium
CBC: Complete blood count
CD61: Monoclonal antibodies (MoAbs) for GPIb expression
CD62P: p Selectin expression
CPD: Citrate phosphate dextrose
DEHP-PVC: Di(2-ethylhexyl)phthalate softener for polyvinyl chloride (PVC)
FITC: Fluorescein isothiocyanate
JC-1: J-aggregate forming cation
LDH: Lactic acid dehydrogenase
MPV: Mean platelet volume
PAS: Platelet additive solutions
PC: Platelet concentrate
PDW: Platelet distribution width
PRP: Platelet rich plasma method
PS: Phosphatidylserine, annexin V marker
PSL: Platelet storage lesions
TCA: Tricarboxylic acid cycle
TOTM-PVC: Tri(2-Ethylhexyl) Trimellitate softener for polyvinyl chloride (PVC)
ΔΨm: Mitochondrial membrane potential

## Footnotes

1 CPD SAGM, Standard Handbags, JP Pharmaceuticals, BR

2 PASIIIM, PAS-E, Macopharma, FR

3 PAS-D, Fresenius Kabi, NLD

4 Sorvall Legend RT centrifugal, Thermo Scientific, US

5 ACS201, Terumo Medical do Brazil, BR

6 Fenwal, Fresenius Kabi, US

7 CDCI3, Indrel, BR

8 AP48L, Presvac, AR

9 LO-Laboroptik GmbH, DE

10 Poch-100iV Diff, Roche Diagnostics Brazil, BR

11 clone VI-PL2, BD Biosciences, US

12 CD62P, AC1.2 clone, BD Biosciences, US

13 CG8, ISTAT Abbott Point of Care, CA

14 Lyte Luminescence ATP detection 1,010,224, Perkin Elmer, NL

15 SpectramaxM5, Molecular Devices Inc., US

16 Vitros 250 Chemistry System, Ortho Clinical Diagnostics, Johnson & Johnson, BR

17 HI 9126, Hanna Instruments Inc., US

18 ApoFlowEx FITC Kit, Exbio, CZ

19 MitoScreen JC-1, BD Biosciences, US

20 FACSCalibur™ and CellQuest™ Pro software, BD Biosciences, US

21 De Novo, Software, US

22 GraphPad Software, US.
