# Supplementary material for: In vitro properties of concentrated canine platelets stored in two additive solutions: a comparative study
Source: BMC Vet Res. 2017 Nov 15;13:334. doi: 10.1186/s12917-017-1236-8 (PMC5688706; doi:10.1186/s12917-017-1236-8)
Supplement: Supplementary file 1 — Composition of the solutions used in the experiment. (DOCX 14 kb) [file 12917_2017_1236_MOESM1_ESM.docx]

| **Supplier** |  | Fresenius Kabi | Macopharma |
| --- | --- | --- | --- |
| **Alternative name** | CPD/Plasma | PAS-D | PAS-E, PASIIIM |
| **Comercial name** |  | Composol | SSP+ |
| **Components** |  | | |
| **Citric acid (annhydous)** | 15.53 | - | - |
| **Sodium citrate (dihydrated)** | 89.59 | - | - |
| **Sodium phosphate monobasic** | 18.52 | - | 28.2 |
| **Dextrose** | 141.82 | - | - |
| **Acetate** | - | 27 | 32.5 |
| **Citrate** | - | 11 | 10.8 |
| **Cl^-1^** | - | 98 | 77.2 |
| **Gluconate** | - | 23 | - |
| **K^+1^** | - | 5 | 5 |
| **Mg^+2^** | - | 1.5 | 1.5 |
| **Na^+2^** | - | 173 | 183.9 |
| **pH** | - | 7 | 7.2 |

**Additional file 1.** Composition of the solutions used in the experiment.
