# Supplementary material for: In vitro properties of concentrated canine platelets stored in two additive solutions: a comparative study
Source: BMC Vet Res. 2017 Nov 15;13:334. doi: 10.1186/s12917-017-1236-8 (PMC5688706; doi:10.1186/s12917-017-1236-8)
Supplement: Supplementary file 2 — Platelet count. pH and CD61 percentage of positive cells mean ± standard deviation. Different lowercase letters represent significantly different values (p < 0.05) between treatments. Different symbols represent significantly different values (p < 0.05) between assessment days. (DOCX 15 kb) [file 12917_2017_1236_MOESM2_ESM.docx]

| **Storage time** | **Day 1** | | | **Day 5** | | | **Day 9** | | | **Day 13** | | |
| --- | --- | --- | --- | --- | --- | --- | --- | --- | --- | --- | --- | --- |
|  | Plasma | SSP+ | Composol | Plasma | SSP+ | Composol | Plasma | SSP+ | Composol | Plasma | SSP+ | Composol |
| **Platelet count (×10^10^/unit)** | 7,197 | 8,13 | 7,8 | 7,23 | 8,18 | 8,16 | 7,17 | 8,09 | 8,24 | 7,35 | 7,8 | 8,17 |
|  | ( ± 2,87) | (± 1,97) | (± 2,5) | (± 3,37) | (± 2,14) | (± 2,64) | (± 3,4) | (± 2,01) | (± 2,72) | (± 3,04) | (± 2,24) | (± 2,91) |
| **pH (22°C)** | 7,162 a§ | 6,73 b§ | 6,44 c§ | 6,1 a§ | 6,36 b§ | 6,04 c§ | 5,48 a¥ | 6,49 b¥ | 5,99 c¥ | 5,42 a¥ | 6,6 b¥ | 6,13 c¥ |
|  | (± 0,24) | (± 0,19) | (± 0,46) | (± 0,59) | (± 0,15) | (± 0,67) | (± 0,34) | (± 0,33) | (± 0,67) | (± 0,20) | (± 0,44) | (± 0,78) |
| **Viable cells / CD61 (%)** | 91,86 | 91,97 | 92,42 | 93,93 | 91,71 | 92,5 | 86,59 | 93,16 | 92,4 | 92,5 | 92,93 | 93,63 |
|  | (± 4,86) | (± 3,68) | (± 4,82) | (± 3,12) | (± 6,71) | (± 4,21) | (± 26,18) | (± 4,59) | (± 5,30) | ( ± 3,06) | (± 5,37) | (± 3,81) |

**Additional file 2:** Platelet count. pH and CD61 percentage of positive cells mean ± standard deviation. Different lowercase letters represent significantly different values (p <0.05) between treatments. Different symbols represent significantly different values (p <0.05) between assessment days.
