# Supplementary material for: In vitro properties of concentrated canine platelets stored in two additive solutions: a comparative study
Source: BMC Vet Res. 2017 Nov 15;13:334. doi: 10.1186/s12917-017-1236-8 (PMC5688706; doi:10.1186/s12917-017-1236-8)
Supplement: Supplementary file 3 — Glucose consumption mean ± standard deviation of platelet concentrates stored in plasma and additive solution for 13 days. Different lowercase letters represent significantly different values (p < 0.05) between treatments. Different symbols represent significantly different values (p < 0.05) between assessment days. (DOCX 13 kb) [file 12917_2017_1236_MOESM3_ESM.docx]

| Period of glucose consumption | Glucose consumption (µmol/day/ 10^10^plts) | | | |
| --- | --- | --- | --- | --- |
|  | 100% Plasma (n=13) | SSP+ (n=13) | Composol (n=14) | Consumption/day |
|  |  |  |  |  |
| Day 1-5 | 24.48 ± 12.82 | 11.12 ± 5 | 10.6 ± 5.13 | 15.93 ± 11.05 § |
| Day 5-9 | 14.55 ± 8.71 | 1.04 ± 2.97 | 3.07 ± 3.69 | 6.14 ± 8.14 ¥ |
| Day 9-13 | 2.9 ± 3.71 | -0.13 ± 0.18 | 0.25 ± 1 | 0.99 ± 2.54 £ |
| Consumption/day/group | 14.65 ± 13.24 a | 4.01 ± 6.07 b | 4.64 ± 5.7 b |  |

**Additional file 3.** Glucose consumption mean ± standard deviation of platelet concentrates stored in plasma and additive solution for 13 days. Different lowercase letters represent significantly different values (p <0.05) between treatments. Different symbols represent significantly different values (p <0.05) between assessment days.
