# Supplementary material for: In vitro properties of concentrated canine platelets stored in two additive solutions: a comparative study
Source: BMC Vet Res. 2017 Nov 15;13:334. doi: 10.1186/s12917-017-1236-8 (PMC5688706; doi:10.1186/s12917-017-1236-8)
Supplement: Supplementary file 4 — Lactate production mean ± standard deviation of platelet concentrates stored in plasma and additive solution for 13 days. Different lowercase letters represent significantly different values (p < 0.05) between treatments. Different symbols represent significantly different values (p < 0.05) between assessment days. (DOCX 14 kb) [file 12917_2017_1236_MOESM4_ESM.docx]

| Period of lactate production | Lactate production (µmol/day/10^10^plts) | | | |
| --- | --- | --- | --- | --- |
|  | 100% Plasma (n=13) | SSP+ (n=13) | Composol (n=14) | Production/day |
|  |  |  |  |  |
| Day 1-5 | 4.5 ± 1.41 | 3.16 ± 1.75 | 2.27 ± 1.62 | 3.28 ± 1.81 § |
| Day 5-9 | 3.66 ± 2.78 | 0.26 ± 1.31 | 1.11 ± 1.23 | 1.70 ± 2.36 ¥ |
| Day 9-13 | 0.4 ± 1.27 | 0.54 ± 0.84 | 0.42 ± 0.67 | 0.45 ± 0.93 £ |
| Production/day/ group | 2.85 ± 2.6 a | 1.37 ± 1.89 b | 1.26 ± 1.43 b |  |

**Additional file 4.** Lactate production mean ± standard deviation of platelet concentrates stored in plasma and additive solution for 13 days. Different lowercase letters represent significantly different values (p <0.05) between treatments. Different symbols represent significantly different values (p <0.05) between assessment days.
